# Future-Oriented Science Learning and its Effects on Students’ Emotions, Futures Literacy and Agency in the Anthropocene

**DOI:** 10.1007/s11165-024-10213-1

**Published:** 2024-11-09

**Authors:** Jessica Chan, Sibel Erduran

**Affiliations:** 1 Plymouth Institute of Education, Rolle Building, Drake Circus, University of Plymouth, Plymouth, PL4 8AA UK; 2 Department of Education, University of Oxford, 15 Norham Gardens, Oxford, OX2 6PY UK

**Keywords:** Agency, Emotion, Climate change, Future-oriented learning, Futures literacy, Informal or non-formal learning

## Abstract

Science education bears the broader objective of nurturing students today to be scientifically-literate citizens of tomorrow who are able to foresee challenges, invent solutions and make responsible decisions for global issues. As a prelude to the new focus of agency in the Anthropocene, this paper presents an intervention on climate change with upper secondary students in a museum of natural history in England. Instructional strategies such as infusing scenarios and arts into scientific discussions were adopted to induce imagination, future-oriented thinking and emotional responses. Statistical results showed that the intervention significantly enhanced participants’ futures literacy, environmental agency and positive emotions. However, it did not increase their interests in learning science in out-of-school context. Implications of this study will shed light on futurising science and climate education in research and practice.

## Introduction

Developing young people’s skills for addressing environmental challenges has become the latest agenda in many science education programmes (Cheung, 2024; Harris & Ballard, 2021; Hadjichambi et al., 2023). The PISA 2018 results showed that as high as 79% of the 15-year-olds in participating countries are aware of climate change. Consistent with the youth environmental activism, 66% thought looking after the environment is important to them. The other side of the coin is that young people also express a sense of hopelessness about global warming (White et al., 2023). Their despair provides a compelling reason for science educators to prioritise student agency and capabilities for dealing with future challenges in addition to scientific literacy.

At an international scale, agency through science learning will soon be measured. PISA 2025 will introduce a new dimension, “sustainability and environmental education” in the science framework (OECD, 2023). For the very first time, student self-efficacy and agency towards socioecological crises gain focus. In good timing with the new tide which will likely receive considerable attention, the study reported in this paper serves as a prelude to investigating how young people relate themselves to environmental issues after taking part in an innovative climate education programme. It aimed at developing students’ future-oriented skills, environmental agency and emotions, measured in six dimensions about participants’ engagement and attributes. To this end the study will add to the knowledge base of how science education can be a catalyst for non-cognitive or generic attributes through purposeful pedagogical design and leveraging the potentials of learning in out-of-school contexts.

## Literature Review

### Teaching and Learning in and out of School

The call for future-oriented science learning should be construed with a set of issues flagged by the science education community. Despite an urge to developing higher-order skills, pedagogical innovations for improving students’ critical thinking or argumentation in primary and secondary science classrooms remain uncommon (Erduran et al., 2019; Vincent-Lancrin et al., 2019; Zeidler, 2016). Although science teachers believe that science education should be more connected to the world outside the classroom so that students understand why science is an indispensable part of everyday life (Oxford University Press, 2022), the way science is taught in school has long been criticised for detaching teaching from other imperative and integral educative goals such as inquiry and problem-solving skills, citizenship, agency and a sense of commitment to future challenges (Alzen et al., 2023; Gutiérrez & Barton, 2015; Schwartz et al., 2023; Thomas & Boon, 2023). In a recent survey of science teachers from 22 countries, only 31% agreed that “current science education is fit for the future”. Worse, 30% of the teachers disagreed that the science curriculum currently taught in their schools “prepares young people to address the challenges that work world will face in the future”, with another 24% neither agreeing nor disagreeing (Oxford University Press, 2022). Overall, the majority of the surveyed teachers believed that science education in its current shape is “not fit for the future” (p. 5). For example, excessive focus on content knowledge in the curriculum may deter students from developing competence in science, which is the ability to identify questions, draw evidence-based conclusions and make decisions based on an understanding of the impact of science on the environment and on society (European Commission, 2019; Schwartz et al., 2023).

Apart from curriculum design, teachers also identified systemic, institutional, financial and conceptual barriers in environmental education (Khalidi & Ramsey, 2020; Ntona et al., 2023). With these perceptions, it is unsurprising that science teachers may shy away from teaching contentious issues pertinent to anthropogenic crises (Alkaher & Carmi, 2024; Monroe et al., 2019). Such hesitations and pessimism that prevail at the frontline are alarming and should not be taken lightly. Teachers are key stakeholders in the education sector. Their beliefs have direct impact on curriculum enactment and how a lesson is organised (Chan & Erduran, 2023; Edwards et al., 2019; Kaufman et al., 2024; Liou et al., 2019; Liu & Zhang, 2024). If teachers are unconvinced about how science education can prepare students well for the future, any grand curriculum objectives and well-meaning initiatives will be at stake.

Furthermore, science learning does not only happen in school (OECD, 2019a). Advancement in technology has blurred the distinctions between learning in school and non-school settings (OECD, 2020). For school-aged children, learning outside formal settings can consolidate what is taught in school (Tang & Zhang, 2020; Tsybulsky, 2019). The positive impacts of non-formal learning contexts extend to psychological and motivational factors such as students’ self-efficacy and career aspiration in STEM (Kirchhoff et al., 2022; Maiorca et al., 2021; Tang & Zhang, 2020). Utilising learning opportunities across contexts prompt students to apply ideas, questions and understandings built within the classroom to authentic situations in their out-of-school lives. In other words, learning experiences in the real world give meaning to what is taught and presented in the classroom, making learning more coherent, meaningful and alive for students. These benefits can be amplified if classroom activities are supplemented by purposeful learning design in non-formal settings (Halverson et al., 2024; Xie et al., 2023). Nonetheless, this field is “a largely uncharted territory” (OECD, 2022). There is substantial research on non-formal or informal learning predominantly focuses on adult or vocational education (Aceron, 2018). More research on school-age students’ learning in out-of-school settings an important area to focus on especially in relation to undertakings that are designed around specific subjects or skills. There is therefore a pivotal part for the scientific community to play in incorporating science education with non-formal education (Ferholt et al., 2020), and investigating the benefits associated with extra-curricular learning opportunities.

### Future-Oriented Thinking and Agency

Perhaps “21st century skills” as a ubiquitous topic has inspired educators to also adopt “futures literacy” from the field of futures studies to delineate students’ competence or learning targets that will help them to navigate the future (Laherto et al., 2023a). Futures literacy is defined as “the capacity to explore the potential of the present to give rise to the future” (Miller, 2007, p. 347). It involves using anticipation to understand and act in a complex emergent context (Miller, 2015). Terminologically, “futures” in plural form highlights the assumption of possible alternative and multiple scenarios which concurrently suggests why foresight, imagination and anticipation are conducive to futures thinking (Miller et al., 2018). Futures literacy originated from a focus on the process of and capacity to anticipate at institutional level with the aim of improving leadership in organisational studies (Miller et al., 2018). As it evolved, more researchers have adopted the term as a universal literacy in wider fields (Facer & Sriprakash, 2021). Although what futures literacy constitutes in learning, its relevance to and application in education are still in an inception stage (Karlsen, 2021; Poli, 2021), this state-of-the-art concept has already drawn interest from researchers in education, more noticeably at tertiary level (Bol & de Wolf, 2023; Kazemier et al., 2021). Emerging evidence suggests that future-oriented thinking, a capability that falls within futures literacy, can positively influence academic performance and engagement in learning (Bol & de Wolf, 2023; Pawlak & Moustafa, 2023). What can be expected is that concept-proof research in futures literacy or future-oriented thinking, particularly its significance in students’ generic skills and other related competencies will likely be the next bandwagon.

Future-oriented thinking enables one to make predictions about the future by analysing, evaluating and reflecting on past and present conditions (Mangnus et al., 2021). Over the last few decades, rapid social, demographic and technological changes have revolutionised our ways of living, brought new inventions and not least widespread problems. In education, these irreversible changes have by far outpaced the speed and capacity of many school or system reforms. As a result, a serious time lag has emerged between the kind of learning that is fostered in many classrooms and the position students should take to envision their desirable future. More alarmingly, the extent to which students are affected by this lag is not known (OECD, 2020). If science education failed to fill students with a sense of commitment and the capabilities for constructing future possibilities, the consequences would be dire. It would be hard to assure future citizens that they can counteract increasingly unpredictable challenges incurred by artificial intelligence, pandemic or socioeconomic inequality. These pressing problems have already caused sweeping effects on all walks of life, and will continue to be the case in the foreseeable future.

Becoming the citizens of tomorrow with a future-oriented mind means students should see the interrelations between their own future, the future of their immediate surroundings and that of the world in distance (Levrini et al., 2021). Ideally, the lag between learning in school and the empowerment students need for inventing their future can be alleviated by a curriculum that is responsive to societal changes while positioning students at the centre. However, student voice is often excluded in curriculum development (Cook-Sather, 2020; OECD, 2020). In fact, the processes of education reform and policymaking often attract criticisms for being opaque and not transparent (Andersen et al., 2009; Fernandez et al., 2008). Intended effects of well designed curricular can be discounted by various types of barriers during implementation. For instance, the UNESCO vision of integrating Education for Sustainable Development into policy and school curriculum, which emphasises young people as agents of change and action, is greeted by practical difficulties in different countries (UNESCO, 2020). Although 40% of its surveyed teachers feel confident about teaching the cognitive aspects of climate change, only 20% of them think they are able to explain how to transfer those educative objectives into practice (UNESCO, 2020). Misalignments between curriculum goals, practice and learning outcomes are commonplace. Evaluation studies on the impacts of curriculum on students’ well-being are also minimal (OECD, 2020). In short, a pioneering step to futuristic science education should establish the relationships between future-oriented curriculum, student participation and key competencies such as future-oriented thinking.

Student agency is a core construct in futures literacy (Häggstörm & Schmidt, 2021) and is highlighted in cogent and far-reaching policy documents (OECD, 2020, 2022, 2023; UNESCO, 2023, 2024). Student agency is not new in research but it has not drawn adequate attention in policymaking until recent years. The Learning Compass 2030, published by the OECD (2019a), sets out “the types of competencies today’s students need to thrive in and shape their future” (p.8). In order to nurture students who can navigate the future, the framework places enormous emphasis on student agency, defined as “the capacity to set a goal, reflect and act responsibly to effect change. It is about acting rather than being acted upon; shaping rather than being shaped; and making responsible decisions and choices rather than accepting those determined by others” (p. 32). Signalled by attributes such as intentionality, fore-thinking, active participation and self-regulation, student agency is both a learning goal and an educational outcome (Code, 2020; Cook-Sather, 2020). Taking these views, activity design and strategies should strengthen agency, which concomitantly is a vehicle for students’ motivation, self-efficacy, sense of purpose and action, and a growth mindset (Doyle, 2015; OECD, 2019b; Rap et al., 2022). Its derivative, “agency in the Anthropocene” (OECD, 2023), is a competency of “being and acting within the world that position people as part of (rather than separate from) ecosystems, acknowledging and respecting all species and the interdependence of life” (White et al., 2023, p.7). This competence that encompasses sustainability, ecological awareness and agency will take the spotlight of the PISA 2025 science framework.

Empirical evidence has supported the role of science education in developing students’ sense of agency and shaping their future (Clarke et al., 2016; Levrini et al., 2021; Martin, 2016). While student agency has become an education goal in many countries, the OECD data showed that it is embedded in only between 0 and 20% of science curricular, compared to 24–80% in subjects of humanities and national language (OECD, 2020). This stark disciplinary contrast attests to our argument that student agency is generally not emphasised or underlined in science education despite it being a key subject from primary to secondary levels. Pedagogically, it is difficult to cultivate agency in conventional or teacher-centred classrooms (Doyle, 2015; Martin, 2016; Rap et al., 2022). Encouraging student agency or autonomy incurs some degree of pedagogical risk-taking and skillful classroom management from the teacher (Chan & Erduran, 2023; Clarke et al., 2016; Edwards, 2023). Also, developing agency in the Anthropocene requires systems thinking and interdisciplinary perspectives but neither of these are advocated by science lessons in school (Melton et al., 2022; White et al., 2023).

### Interdisciplinarity for Science Education

An interdisciplinary approach is paramount to higher-order and future-oriented science learning (Convertini, 2021; Fooladi et al., 2023; Jafari & Meisert, 2021; Melton et al., 2022). Other than cognitive skills, evidence suggests that the benefits of incorporating interdisciplinarity in science learning also include students’ emotional competence (Gao et al., 2021; Herman et al., 2020; Johnson & Czerniak, 2023). The relationship between science, technology and other forms of epistemic praxis is crucial to a better understanding of future and global challenges (Ferholt et al., 2020; Zeidler & Sadler, 2023). In the classroom, interdisciplinarity can be augmented through activities (e.g., Chan & Erduran, 2022) that guide students to collaborate, transfer concepts and identify commonalities between different disciplines (Aurava & Sormunen, 2023; Fooladi et al., 2023; OECD, 2019a). These strategies are the exact ingredients for developing 21st century skills (Pellegrino, 2017).

### Rationale for the Study

The lapse between the current state of science education in school and the kind of competence imperative to students and the society at large entails a turnaround in how science should be taught. Yet systemic reform requires long-term planning, hefty resources and mobilisation, and the smallest impact may take years to be felt. Small-scale innovations underpinned by future-focused learning goals in non-school settings is a viable and measured step to testing the water, gathering evidence and casting new possibilities. Hence, this study embarked on futurising science education by testing the effectiveness of innovative pedagogy in an out-of-school context in realistic circumstances where students can attend a local museum for out-of-school activities. The guiding question is “Does future-oriented science learning induce students’ environmental agency, futures literacy, emotions and interests in interdisciplinary learning outside school”? The following sections will describe how we engineered this intervention with a museum.

## Methodology

### Context and Design

This study was part of the FEDORA project (https://www.fedora-project.eu) conducted in four European countries with an overarching aim of futurising science education (Cassini et al., 2023; Erduran & Ioannidou, 2021; Ioannidou & Erduran, 2022; Laherto et al., 2023b). The resources used with students (Erduran & Ioannidou, 2023) were refined in the context of a sub-project called Project FutuRISE and they are freely available to download at the following link: https://www.education.ox.ac.uk/wp-content/uploads/2024/06/Future-Oriented-Learning.pdf. The intervention with high school students were conducted in two phases over the course of a year. The first round aimed at undertaking a pilot as well as building relationships with stakeholders in the local contexts. Feedback was collected in the first round, based on which the teaching materials and activities were revised before the second-round implementation, for example, by postulating and verifying the hypothesis between experiences of science learning and emotions (Gao et al., 2021; Membiela et al., 2023; Riffert et al., 2021; Tytler & Ferguson, 2023; Vilhunen et al., 2023). This timescale also allowed us to capitalise on some positives of design-based research such as iteration, evaluating and revising the intervention, and reflexivity of the researchers between the two stages which in turn strengthened internal validity and reliability of this study (Hoadley & Campos, 2022).

Before designing the intervention in England, the research team formed an open-schooling network by linking up researchers, museum experts and teachers of science. After various meetings and a short questionnaire to prompt collective thinking, this network of enthusiasts produced some sensible suggestions for translating the objectives into tangible action plans to be rolled out in ways that would work effectively with the context in England. With the network support as point of departure, the research team co-designed the content of the intervention with the science teachers from a local secondary school. Both parties agreed on choosing climate change and sustainability as the topic so the intervention was titled “Climate change and the future of learning”. At the same time core elements such as facilitating interdisciplinarity, student agency and future-oriented skills permeated every part of the design. For instance, imagination is higher-order thinking and is conducive to scientific and epistemic thinking, creativity and learner agency (Kaag, 2014; Tateo, 2015; Zittoun, 2022); and the use of scenarios in science teaching can prompt imagination and future-oriented thinking (Barelli, 2022; Levrini et al., 2021; Miller, 2007). Our intervention tasks thus included break-out groups that would guide students to imagine different future scenarios as consequences of climate challenge. To reinforce agency in the Anthropocene and make the session more relatable, the activities also directed students to link future scenarios to their behaviour in everyday life, as well as to personal plans for the future (Drymiotou et al., 2021; Häggstörm & Schmidt, 2021; Levrini et al., 2019, 2021; OECD, 2018, 2023; Skamp et al., 2019).

Interdisciplinarity is a prime characteristic of the intervention as characterised by other work of such nature (Chan & Erduran, 2023; Erduran et al., 2019; Ferholt et al., 2020) so a great variety of atypical teaching materials were used. These ranged from artworks, poems written by English poets, published personal diaries, historical objects to policy reports. These materials were selected as stimuli to aid students’ conceptual understanding, imagination and connecting science to other disciplines alongside the presentation of hard data about global warming. The purpose was to render a multi-faceted sense of affective feeling behind those statistics and numbers and encourage students to explore climate change as socioscientific problems more complicated than an exclusive science topic situated in a vacuum. Consequently, students would be able to develop fresh thinking on this widely debated crisis by examining scientific arguments through an interdisciplinary lens.

Infusing literature and art and connecting personal reflections to the future were designed to also test any emotional or agentic responses (Barelli et al., 2022; Oliveira et al., 2015; Skamp et al., 2019; Vilhunen et al., 2023). To bolster students’ imagination, an array of future scenarios was discussed, in which the future could be projected through different possibilities (Burden & Kearney, 2016; Paige & Lloyd, 2016). This was a mindful and clear message conveyed to all participants to eliminate any deterministic interpretation of the future fuelled by the common apprehension about global warming, and in turn to implicitly encourage them to form agentic beliefs about one’s own position and plans out of the broader parameters.

The intervention sessions, co-run by museum educators and the research team, were conducted in a Natural History Museum. The museum is a 160-year-old establishment curating a collection of seven million objects and 30,000 zoological type specimens. Other interactive and personalised activities included a museum tour, individual time for exploring biodiversity which was the then flagship event of the museum, and hands-on experiences where students could have detailed and manual inspection of specimens of extinct insects. In summary, the embedded learning goals were enabling student voices (Cook-Sather, 2020; Rap et al., 2022), imagination (Barelli et al., 2022; Kaag, 2014; Levrini et al., 2021; Zittoun, 2022), future-oriented thinking (Aurava & Sormunen, 2023; Bol & de Wolf, 2023; Mangnus et al., 2021) and agency in the Anthropocene (OECD, 2023; Rap et al., 2022; White et al., 2023).

### Participants and Data Collection

The intervention schedule was divided into two rounds. Feedback from the first stage was adopted to revise the content for the next round. This paper presents results from the second-stage intervention which consisted of three sessions. Each of them lasted three hours and was delivered as a one-off session in the museum to students aged 16–17 (Year 12 in the English system). Recruitment of participants was facilitated by our open-schooling network and the university outreach team. Data collection had been approved by an ethics committee prior to the fieldwork. Students volunteered to take part after receiving an invitation and information about this research. The sample included in this paper is 40 students (16 male and 24 female) from three secondary schools who took part in the second round, i.e. one participating school in any single session. Each participant filled in a questionnaire before and after the session. The questionnaires collected information about students’ prior engagement in the topic, future aspirations, views about science learning and their emotional responses in reaction to the activities.

### Operationalisation, Instrument and Data Analysis

The data source is students’ responses to pre- and post-intervention questionnaires. Both of which were purposefully designed for testing the intervention effects. Since psychometric measurement for student agency is under-developed (Jääskelä et al., 2023), and more so in the case of environmental agency, the instrument was adapted from environmental attitude and action scales in accordance with the age group and specificity of this study (Alisat & Riemer, 2015; Kaiser et al., 2018). Statements indicative of futures literacy were added. The two questionnaires consisted of 42 items in total. Open-ended questions asked about students’ demographic background and comments of the session, whereas multiple-choice and 5-point Likert-scale questions were used for the target variables.

To achieve greater statistical power, Wilcoxon signed-rank test was performed to analyse this paired-sample nonparametric dataset (Elliott & Buttery, 2022; Li & Johnson, 2014; Rosenblatt & Benjamini, 2018). Our analyses focused primarily on students’ perceptual changes in three dimensions, which would evidence some effects of the intervention to futurise science education. These dimensions are views about science teaching, environmental agency, and futures literacy. They were operationalised by a total of 16 Likert-scale items from the pre- and post-intervention questionnaires. In addition to analysing individual responses to each discrete test items within the three dimensions, an aggregated score was also calculated to determine if there is any significant difference in the respective dimension as a whole, i.e. a composite score comparing the pre- and post-medians of all the items which constitute that dimension, and whether the overall statistic has significant value. Responses from a different set of dimensions, collected by either pre- or post-intervention question items but not both, were analysed by descriptive statistics to investigate students’ learning experiences and future-oriented thinking. These three dimensions were not intended for predicting attitudinal changes. Instead they were indicative of emergent responses and orientations especially after the scenario-based activities.

## Results

This section presents results of the six dimensions in our measures of interdisciplinarity, agency and future-oriented thinking. These six dimensions are – (1) engagement with climate change issues; (2) views about science teaching; (3) environmental agency; (4) futures literacy; (5) planned future actions; and (6) epistemically-related emotions. For the three dimensions with either pre- or post-intervention data (Sect. 4.1, 4.5 and 4.6), we present the median rank (Mdn), mode and the number of responses (n). Results of the other three dimensions using the Wilcoxon test will be presented in *z*-score, *p*-value and effect size (*r*), if statistically significant to confirm students’ pre- and post-intervention changes (Sect. 4.2, 4.3 and 4.4).

### Engagement with Climate Change Issues

For baseline information, before the session students were asked to indicate how often they had taken part in activities related to climate change during that school year. Results showed that prior to this intervention, on average they only occasionally participated in this type of activities (Mdn = 2, mode = 2, *n* = 40). Their limited interest suggested low motivation in climate education.

### Views about Science Teaching

We were interested in understanding students’ views on how science should be taught in school, its links with other subjects or outside school contexts, and if their views shifted after the intervention. This dimension was measured by three questionnaire items. Out of the 40 participants (N), 35 pairs of pre- and post- responses were recorded (*n* = 35).

The first item asked if students enjoyed infusing interdisciplinarity in science learning, “I like when teachers link topics in science lessons to other subjects such as humanities, geography and mathematics”. The test result found a significant difference in their attitude after participating in the intervention (*z* = -2.500, *p* = .012), confirming rejection of the null hypothesis. Effect size was calculated, by dividing the *z* value by the square root of the number of observations (Field, 2005; Fritz et al., 2012; Rosenthal, 1991; Rosenthal & Rubin, 2003). In this case it is the number of observations at the two time points (35 × 2), which makes − 2.5/(√70). The intervention has successfully increased students’ interests in an interdisciplinary approach to science learning, with an effect size of *r* = .298 (Cohen, 1988, 1992; Ivarsson et al., 2013; Ricca & Blaine, 2022).

Views about learning in out-of-school settings was measured by the item, “I believe we should have more opportunities to visit museums and other organisations to help us understand what we learn at school”. Result shows that the post-intervention median ranks (*Mdn* = 5) were not statistically significant compared to those prior (*Mdn* = 4, *z* = -1.807, *p* = .071). Null hypothesis cannot be rejected and our intervention did not significantly enthuse students to visit non-school places for science learning.

The third item asked students to what extent they agreed “schools are teaching us the skills needed to deal with future problems such as climate change”. The difference in the median ranks (*Mdn* = 3) was not statistically different (*Mdn* = 3, *z* = -1.099, *p* = .272). Our workshop did not alter students’ judgements of whether schools are preparing them for the future.

To test the overall effect on views about science teaching, a composite score combining all the items was calculated for each student. The aggregated result shows a significant difference between their pre- and post- responses in this dimension as a whole (*z* = -2.395, *p* = .017, *r* = .286). The intervention did modify students’ views of how science should be taught. Precisely they became more interested in an interdisciplinary approach. Table 1 summarises the results.

**Table 1:** Views about science teaching (*n* = 35)

Item	*z*-score	*p*-value	*r*
*I like when teachers link topics in science lessons to other subjects such as humanities*,* geography and mathematics.*	− 2.500	0.012*	0.298
*I believe we should have more opportunities to visit museums and other organisations to help us understand what we learn at school.*	-1.807	0.071	
*I think schools are teaching us the skills needed to deal with future problems such as climate change.*	− 1.099	0.272	
Aggregated (the differences between the composite pre- medians and post- medians)	− 2.395	0.017*	0.286

*p < .05

To further examine an interdisciplinary interest in science, an open-ended question asked for students’ ideas of any non-science subjects that are related to building a sustainable future. Before the intervention, free-writing answers indicated that students were restricted by disciplinary boundary. Their answers usually linked “building a sustainable future” to other STEM subjects such as mathematics and geography. However, their post-intervention answers showed higher interdisciplinary awareness. Below are two examples:

*Art*,* gives individuals information about climate change throughout history and can invoke an emotional response. Geography*,* sociology*,* and history can all teach students about global warming and to educate pupils and encourage to build a sustainable future.*

*Every action has a consequence*,* so all subjects can in some way have a role in building a sustainable future. E.g. Business will link to decisions made that will impact the climate.*

### Environmental Agency

Table 2 lists the results of differences in students’ environmental agency measured by nine statements. Students showed significant post-intervention changes in six of them (*p* < .05); but not in three others. For the six statistically significant items, their effect sizes are between small and moderate and vary only within a narrow range (between 0.228 and 0.342).

**Table 2:** Environmental agency (*n* = 38)

Item	*z*-score	*p*-value	*r*
*Looking after the global environment is important to me.*	-2.530	0.011*	0.290
*I can identify some consequences of climate change.*	− 2.642	0.008*	0.303
*It is important for me to take some actions to limit my impact on global warming.*	-2.540	0.011*	0.291
*It is the responsibility of certain groups (e.g. scientists or politicians) to take actions against climate change.*	− 0.921	0.357	
*I can do something about climate change.*	-2.202	0.028*	0.252
*Personal choices (e.g. consumer behaviour) have an impact on climate change.*	− 0.842	0.400	
*I often reduce the energy I use at home (e.g. by turning off the lights when leaving a room) to protect the environment.*	-1.995	0.046*	0.228
*I often choose certain products for ethical or environmental reasons*,* even if they are a bit more expensive.*	-2.358	0.018 *	0.270
*I often sign environmental or social petitions online.*	− 1.208	0.227	
Aggregated (the differences between the composite pre- medians and post- medians)	− 2.985	0.003*	0.342

*p < .05

Composite pairs from each of the pre- and post- test scores were calculated for the aggregated effect. Results show that overall, our intervention significantly increased students’ environmental agency (*z* = -2.985, *p* = .003, *r* = .342).

### Futures Literacy

Futures literacy was measured by four items, all of which showed significant differences (Table 3). Due to this consistency across variables, the aggregated effect produced a powerful statistical difference (*p* < .001). Effect sizes range from 0.286 to 0.456, achieving small to moderate improvements in students’ futures literacy (Cohen, 1988, 1992).

**Table 3:** Futures literacy (*n* = 38)

Item	*z*-score	*p*-value	*r*
*I can imagine some negative scenarios as a result of climate change.*	-2.496	0.013*	0.286
*I can imagine some positive scenarios as a result of actions against climate change.*	-2.876	0.004*	0.329
*I can identify some steps needed to achieve a sustainable future.*	− 3.740	< 0.001 *	0.429
*I can identify some steps that I*,* personally*,* can take to achieve a sustainable future.*	-3.105	0.002*	0.356
Aggregated (the differences between the composite pre- medians and post- medians)	− 3.978	< 0.001*	0.456

*p < .05

### Planned Future Actions

After the intervention, we gathered data on students’ motivation of acting upon climate change (“after the session I am more likely to …”) as an indicator of their agency oriented to the future (1 = not at all; 5 = very much; *N* = 40). This dimension was not measured in the pre-intervention survey. Hence, descriptive analyses were performed (*n* = 38) given such information can be informative for planning lessons that would motivate students in action about climate change.

Students reported that after the session they would be more likely to “reduce the energy use at home to protect the environment” (Mdn = 4, mode = 4); “choose certain products for ethical environmental reasons, even if they are a bit more expensive” (Mdn = 4, mode = 4); “sign environmental or social petitions online” (Mdn = 4, mode = 4).

Qualitative responses were collected to validate whether and how students found the activities inspiring and useful. Below are two examples illustrating how students linked the intervention directly to their future planning:

*This session was relevant because it gives me an insight into my future and my children’s generations. Finding solutions.*

*It is [relevant] as I would like to go into a career to do with geography which I feel strongly links with climate change & everything that’s been discussed today.*

### Epistemically-related Emotions

At the end, students rated the strength of emotions they had experienced during the activities (1 = not at all; 5 = very much). Since the design was to find out if the intervention provoked any emotions, we did not seek these ratings prior to the session. Descriptive analyses indicated that on average (*n* = 38), students expressed higher level of positive than negative emotions after participation. They felt surprised (Mdn = 4, mode = 4); confused (Mdn = 2, mode = 1); anxious (Mdn = 2, mode = 1); bored (Mdn = 2, mode = 1); frustrated (Mdn = 1, mode = 1) and hopeful (Mdn = 3, mode = 3). Interestingly, their most intense emotion is feeling motivated (Mdn = 4, mode = 5).

## Discussion and Implications

The intervention in our project to futurise science learning through a customised design has produced the strongest effect on participants’ futures literacy. Statistical differences are significant in all of the variables. The aggregated effect size of 0.456 suggests a medium effect of the innovative approach (Cheung & Slavin, 2016; Schäfer & Schwarz, 2019). Futures literacy can be supported by multifaceted and participatory pedagogy (Hadjichambi et al., 2023; Häggstörm & Schmidt, 2021; Levrini et al., 2021), which prompts an awareness of and a capacity for planning and preparing for the future while being open to novelty (Bol & de Wolf, 2023; Miller et al., 2018). Our use of scenarios stimulated students’ imagination and ideas about plausible future against uncertainty. This kind of creative and proactive thinking aroused foresight (Government Office for Science, 2021; Miller, 2007; Rincón & Díaz-Domínguez, 2022) and simultaneously inspired students to evaluate pre-emptive actions (Aurava & Sormunen, 2023; Skamp et al., 2019). Prior to participation, students reported low or inactive engagement in learning about climate change but their motivation and sense of commitment to future actions increased afterwards.

On the other hand, definition of futures literacy, its criteria and measurement are a new field that requires more research. Apparently there are no immanent characteristics or attributes for describing and capturing futures literacy, hence it is deemed as a primitive scientific concept with warning against codification (Facer & Sriprakash, 2021; Karlsen, 2021). The plus side is that it leaves researchers ample freedom and originality to conceptualise this new genre of literacy in science education. Our study has contributed to science education by translating futures literacy into ecological awareness and learner agency. Since future is inextricably tied to and dependent on environmental protection, further research should consolidate the relevance of futures literacy to climate or environmental education in the context of science education.

The “engage and show, don’t just tell” method in future-oriented teaching has evidently motivated and engaged the participants (Bol & de Wolf, 2023, p.4). The three-hour session was primarily structured by student-led activities that emphasised autonomy, creativity, constructing diverse views and group dialogue. Participants were free to choose any museum exhibits or specimens before explaining how the selected objects supported their sensemaking and reflections on the scientific knowledge imparted (Pierson et al., 2023). Scientific data were co-presented with paintings, artworks, historical artefacts and poems. This incorporation of aesthetic experiences has “personalised” science learning, allowing students to invent their own interpretations and decisions taking the subject matter into account. A systematic review of 49 studies by Monroe et al. (2019) found that making learning personally relevant and meaningful, and structuring activities that engage learners are two principles for effective climate change education. Our instructional model and its effects are a testament to these recommendations.

The aesthetic and humanistic elements infused in the activities may have triggered students’ emotional responses (Elliott, 2022). Emotions are inherent in science learning (Siry & Brendel, 2016; Wickman et al., 2022). This study attested to inducing affect such as enjoyment, boredom, anxiety and sense of hope in the process of science learning scrutinised in previous research (Raker et al., 2019; Pierson et al., 2023). Learning about climate change can be emotionally draining and “joy-killing” (Beasy et al., 2023; Russell et al., 2023). Our intervention consisting of arts and multimodal activities successfully sparked positive affect and interdisciplinary thinking. Students are more likely to apply scientific knowledge to real-world problems if they engage in an art-science or interdisciplinary domain (Hannigan et al., 2022). The aesthetic experiences created by the pedagogical invention enabled students to forge meaning for learning about the climate (Herman et al., 2020; Wickman et al., 2022). The new meaning rendered a personal relationship between the students and the learning objectives, which have possibly invoked their sense of agency towards environmental challenges (Membiela et al., 2023). There has been scant research on the role of affect in science education so more empirical work is needed to decipher this intrapersonal aspect of 21st century skills (Pellegrino, 2017).

By positioning young people as agents of change and shaping the future, our study has fostered participants’ environmental agency (Oliveira et al., 2015). Agency is plainly a messy concept and it is also the case in science education (Martin, 2016; Oliveira et al., 2015; Rap et al., 2022). Consensual or scientific measures for the construct of agency are yet to be established but analysis of measurement invariance has validated factors such as competence beliefs, opportunities to make choices, opportunities for influence, self-efficacy and ease of participation (Jääskelä et al., 2023). Using authentic problems incurred by global warming, the semi-structured tasks guided students to decide on their learning materials, back up ideas by scientific knowledge and imagine multiple futures which then ascertained roadmaps for exerting individual influences. Engaging in these activities gave rise to argumentation, creativity, decision making, generating impact and a sense of empowerment. This integrated design substantiated the epistemic, cognitive and non-cognitive aspects of a model for futurising science education. Agency can be bolstered when students are given choices on what and how to learn, and using the learning content for real-life assignments and modifying the contexts (Cuzzolino et al., 2024). The results dissent from the claim that “student agency is not easily accomplished and we should not expect large consequences from relatively small interventions” (Doyle, 2015, p. 277). Our one-shot intervention in a future-oriented configuration is shown to be effective enough for promoting agency. This study also contributes to research on student agency in environmental issues by contextualising it in scientific and museum contexts (Stenalt & Lassesen, 2022).

The positive results of students’ increased interests in an interdisciplinary approach to science learning echo the widely-researched recommendations. Teachers generally also support a cross-curricular method for environmental education (Howard-Jones et al., 2021) but very few programmes have drawn on other disciplines (Monroe et al., 2019), with consequences of fragmented or disjointed understanding of a vastly sociopolitically-nested topic. Unlike those undertakings, our study offers an example of how deploying the arts to complement science learning can motivate students while alleviating apprehensions often inflicted by discussing global warming (Russell et al., 2023).

On the other hand, taking part in this research did not incentivise the participants to seek more informal learning opportunities. It is possible that students studying for A-levels, i.e. the qualification for university entrance in the English system, might not see the immediate benefits of informal learning to high-stakes examinations. In any case, students should be able to “continue to learn about science outside of school” (National Research Council, 2012, p.1). Harnessing the potentials of out-of-school learning requires coordination between adult brokers, programmatic support, access to infrastructure and platform, and inter-contextual knowledge transfer (Dahn et al., 2024; Elliott, 2022). This study presented a case of researchers taking the broker role of connecting school and a museum.

A strength of this study is that instead of using Cohen’s *d*, the calculation of effect sizes adhered to the nonparametric assumptions and accounted for the sample size (Foster, 2023; Fritz et al., 2012; Ivarsson et al., 2013; Taylor & Alanazi, 2023). The small to medium effect sizes achieved by a few one-off sessions on a small participant sample imply that those effects are likely to be stronger if a similar intervention is to be scaled up with a bigger sample (Fritz et al., 2012; Paris & Luo, 2010; Taylor, 2023), and possibly more pronounced if splitting the intervention into a series of sessions over a period of time (Kraft, 2020, 2023; Ugille et al., 2014). Taylor et al. (2018) has meta-analysed the effect sizes from 96 studies in science education and calculated a grand mean of 0.489. Several of our variables have effect sizes either exceeding or close to this benchmark. Considering our short-lived teaching delivered to a small student group, the resulting effects are arguably powerful and shed an invaluable light for further research. Directions include replicating the pedagogical features to a range of student characteristics by age, gender, motivation and country; or in different contexts both within and outside school. The focus on students’ attitudinal and affective factors relying on self-reported data is a limitation of this study. Subsequent research on future-oriented learning can include cognitive variables such as subject knowledge and reasoning, and doing so using different methods including standardised tests.
